# Editorial and Miscellaneous

**Published:** 1856-02

**Authors:** 


### EDITORIAL AND MISCELLANEOUS.

### "MEDICAL REFORM."

One of the most significant facts to which our attention has been directed of late, is one announced, in a late article from the Buffalo Medical Journal, which we find in the January number of Reese's American Medical Gazette.

We could wish that the view there presented of the tendencies and associations of quackery, in its multiplied forms, might receive the attention, not only of the profession, but of every intelligent and moral mind in the land, believing as we do, that a consideration of the subject would be sufficient to induce every virtuous individual to turn with disgust from the labors and teachings of these *new lights* in morals, as well as in medicine. The particular fact to which we alluded in the outset, is the designation of that well known and infamous *abolition* organ, the "New York Tribune," as a source of more evil to the profession, and support to quackery, than any or all the attacks of our open enemies. Never losing an opportunity to traduce the fame of the true physician, it has ever been the organ of quackery. The advocate of Spiritualism, Mesmerism, Fourierism, Free Love; and especially of the Hydropathic and Homœopathic delusions; it has never stickled to puff any other form of humbug. For a time, it was the mouth-piece of Chronothermalism, and, inconsistent to the last degree in its advocacy of antagonistic theories, it has only been consistent in its undisguised attacks on the true physician.

From the radical and gross errors of their pseudo science, we find them asserting (according to Dr. Hunt,) that a "marriage becoming distasteful to either of the parties, it is the duty of that party to dissolve the connection, and enter upon a new one, with some more congenial spirit, that the offspring of unhappy marriages are, *physiologically*, imperfect, liable to early death; or, living, to become the prey of disease or passion. Not only this, but that such a marriage entails ill health and suffering on the parties themselves, and from this stand-point, an attack is made—not on hasty, ill-considered or unnatural matches; but on the institution itself, on the Bible, as recommending it, and on the legitimate profession of medicine, as the well known opponent of licentiousness."

After giving a list of some of the most prominent and infamous of these medical and social reformers, among whom we find a *female doctor* from Boston, rendered notorious, by presiding over a convention to discuss woman rights and Spiritualism, the editor of the Buffalo Journal remarks, that "in the so-called medical works of these writers, may be found the impersonation of all heresy and schism; of hatred to that medical profession which it cannot bend to its doctrines; of advocacy of Water Cure, Homœopathy and Spiritualism, of a monstrous and spurious physiology, whose insane teachings lead its followers over the ruins of the social fabric, morality, and religion; of a debasing sensualism, which, in the name of friendship to the gentler sex, would sink them to the lowest degree of degradation."

Such, we are assured, are the doctrines and teachings to be found in the therapeutics of Hahneman and Preissnitz, respectively, the great heads of Homœopathy and Hydropathy; "the text books of the former being filled with stupid transcendentalisms, which befog and prepare the mind for that farrago of nonsense to be found in the writings of the Spiritualists and Communists, that balderdash about "higher harmonies," "marriage of the affinities," and "passional attraction." The very nosology of Hahneman making vice a disease, and a "disposition to lie," or "steal," or "murder," to be cured by "thirtieth potencies," and in this point of view, as the Editor very appropriately remarks, it would be well to confine the practice of Homœopathy to penitentiaries and jails.

And yet more, "however wicked may be the tendencies of the infinitessimal delusion—however great an upsetting of all power to discriminate between right and wrong, the mere adoption of such a theory must imply—it is in the ranks of Hydropathy, that rascality has attained to its utmost development," most of the water cure lecturers and physicians in the charge of cures—a number of whom are mentioned—"are also the shameless teachers of doctrines, so infamous, that even the liberal use of language permitted in medical writing, is insufficient to describe their enormity; doctrines which would make all women concubines, all property plunder, all religion an irresponsible pantheism, and would constitute the 'higher law' of the seared conscience of the adulterer, the only guide to 'right and wrong,' it being to the upholders of this worse than barbarian creed, that wives and daughters are committed, when sent to 'water-cures' for restoration."

But the great point which stands prominently forth in this matter, is that "between all the various forms of quackery, and those of religious and social infidelity; there is such a bond of affinity, that we find the same class of minds attracted by both those who daintily tamper with the one, are extremely liable to be entrapped by the other—he who admits the propriety of female physicians, asserts female equality; believing this, he is bound to concede to the equal female, her equal rights, and in doing this, he does away with marriage, for in Christian marriage, ordained by God, there is, and can be, no equality; *Facilis descensus Averni.* The weak chain of reasoning thus traced, has beguiled many a feeble mind."

We close our extracts from Dr. Hunt's article, with his remark, that "the truly just and conservative position of the medical profession proper, was never more forcibly impressed upon us, than by the remark of a friend, that all 'medical reformers,' so far as his acquaintance—a large one—extended, were men of low morality.

"The honest seeker after truth in medicine, finds field enough for labor in the furtherance of reform in the treatment of disease, to be attained by steadfast study and effort, not by abuse and detractions of those who have labored before him." These are the sentiments of those who are in the midst of the hot-bed of all the corrupting innovations of the day, and have the best opportunity of knowing, whereof they affirm, the clear tendencies of which, are to destroy science, and sap the foundations of our social structure; and the fountain from which this stream of moral poison constantly issues, is the press of Fowler & Co., New York, whose bare names, upon any publication, should seal it, to every lover of truth and right.

### ATLANTA MEDICAL SOCIETY.

We shall commence in the next number of our Journal, reports from the Secretary, of the most interesting cases of disease, presented to the Atlanta Medical Society. This, we think, will be an interesting and profitable feature, and we would suggest to other Medical Societies, among the members of which, we have a circulation, that they would confer a favor upon us, and contribute to the advancement of science, by reporting, through their Secretaries, such portions of their proceedings as might be supposed to be interesting to the profession generally.

Our local Societies should be efficient instruments in the advancement of medicine, and we would take the liberty of urging upon the profession—with whom we are in intercourse through this medium—the importance of organizing, not only in the towns and villages, but even in the country, where a half dozen physicians can be found sufficiently contiguous to enable them to meet once a month.

We presume there are few true medical men, at the present day, who do not appreciate the value of such associations, not only as a means of improvement to themselves, and as affording an opportunity for social intercourse with professional brethren, but, also of discharging their obligations in contributing to the upbuilding of legitimate medicine by the results of their experience, and at the same time, erecting one of the most effectual barriers to the inroads of quackery, in both its regular and irregular forms. We are much pleased at the decidedly increasing interest which is manifested in the weekly meetings of the body, whose name heads this article, and confidently anticipate valuable aid, in filling our pages, from their records.

---

### THE CINCINNATI MEDICAL OBSERVER.

The first number of this Journal was issued in January of the present year, under the editorial management of George Mendenhall, M. D., John A. Murphy, M. D., Professors of Obstetrics and Materia Medica in the Miami Medical College, and Edward B. Stevens, M. D. The first number is neatly executed, and is almost entirely filled with original communications.

From the tone of the editorial department, as well as from an acquaintance of several months with one of the Editors, (Prof. John A. Murphy,) who we know to be a gentleman of high professional attainments, we bespeak for this Journal an elevated position among the Medical Periodicals of our country. With our best wishes for its success, we, with pleasure, add it to our list of exchanges.

---

### TILDEN & COMPANY'S EXTRACTS.

We hereby acknowledge the receipt of a number of the solid and fluid extracts of Messrs. Tilden & Co., not having had time to speak of their merits, from experiment, we insert notices of them from two Journals of high character, and shall recur to the subject when we have had an opportunity of testing their virtues—accompanying the present number of our Journal, we send out the advertisement of Messrs. Tilden & Co., from which all the particulars can be obtained:

"*Tilden & Company's Extracts.*—We have just received from the manufacturers, Tilden & Co., of New Lebanon, New York, a variety of their extracts, comprising twenty-four bottles of fluid extracts, and twenty-one of inspissated alcoholic and hydro-alcoholic extracts. These preparations embrace many of the most important vegetable remedies employed by the medical man, a large proportion of them being prepared from indigenous plants. Many of the inspissated extracts are officinal, while the fluid extracts—an elegant form for administering medicines—are just coming into use, but very few of them having been introduced into the last edition of the *Pharmacopœia.* We very much mistake, however, if they do not eventually take the place of the most of the preparations now in use.

"These preparations are particularly well adapted to the wants of the country practitioner, on account of their concentrated form, as well as their agreeable taste.

"For further particulars, we refer to the advertisement of Tilden & Co., and to a notice of their extracts, taken from the *American Journal of Pharmacy*, which will be found in another part of this number. We shall embrace opportunities of using the extracts, and of recording our experience with them".—*N. J. Medical and Surgical Reporter.*"

"*The Fluid Extracts of Tilden & Co.*—In our last number we acknowledged the reception of twenty-one specimens ot Tilden & Co.'s fluid extracts, then too late for notice. Since that time, two months, they have been kept in a closet at the temperature of the store, with the mercury varying from 70° to 94°, with no means taken to prevent its influence. These preparations are said to be made according to the *Pharmacopœia*, when officinal, and in several instances, according to formulæ, published in this journal when not recognized by authority, whilst some are made by the judgment of the manufacturers, always, in such caaes, being of the strength of 1 oz. to f℥j. They are put up in pannelled bottles, holding about four fluid ounces, labelled and wrapped.

"*Balladonna, Hyoscyamus, and Lobelia.*—These fluid extracts may be noticed together. They are, when shaken, dark-green fluids, owing to the chlorophylle they contain, the first two appearing evidently to be made from the recent plant. The belladonna and hyoscyamus smell strongly of the recent plants when bruised, and have their peculiar taste well developed. Although made from the green plants, the proportion is a fluid ounce to an ounce of the dried plant, which is ascertained by drying a small quantity of the herb, and ascertaining its loss, and then using a corresponding quantity of the recent plants. Being concentrated in vacuo, and preserved with a little alcohol, these fluid extracts appear to be fully charged with the sensible and medicinal properties of these drugs.

"*Rhubarb, Senna, Rhubarb and Senna, Senna and Taraxacum, Senna and Spigelia, and Buckthorn.*—These appear to be well made. The rhubarb, while it has the proper odor and taste, is rather more fluid than it usually appears. So much depends on the choice of the root, that it is a difficult matter, obscured as the taste is with aromatics, to judge with certainty. The senna is excellent, and is all the better for being made in vacuo—as are the compound extracts containing it, spigelia, taraxacum, and rhubarb. They are all preserved with sugar, and have a syrupy consistence. The fluid extract of buckthorn is made from the nearly ripe berries, and presents the form of a dense reddish-brown syrup, from which a portion of the sugar has crystallized, owing, probably, to the alcohol added to keep it. Its activity is such that a teaspoonful is given as a dose.

"*Serpentaria, Chamomile, and Gentian.*—There are two formulæ for fluid extract of serpentaria—that of Alfred B. Taylor, made with diluted alcohol, of the strength ℥j to f℥j, and that of John C. Savory, preserved with sugar, and of the strength ℥ss to f℥j. The latter appears to be that followed by Mr. Tilden. The fluid extract, however, does not possess the decided bitterness and camphorous pungency that it should, nor is its aroma as well marked as it should be. The chamomile represents only the bitter extractive, as none of the aroma of the flowers is perceptible. Although difficult to retain, when water is the menstruum, a preliminary tincture with alcohol, to be evaporated afterwards spontaneously, and added to the concentrated infusion, would insure the presence of the volatile portion to a considerable extent. The gentian is well prepared.

"*Cimicifuga, Scutellaria, Buchu and Uva Ursi*, well represent their respective drugs. The scutellaria has recently been tried by Dr. Bates with marked success in nervous diseases. The aroma of the buchu speaks for itsslf.

"*Sarsaparilla, Compound Sarsaparilla, Stillingia, Rumics Crispus and Taraxacum.*—The fluid extract of sarsaparilla is not the officinal preparation, nor is the compound extract made by that recipe, but contains conium—the simple extract is, probably, made from American sarsaparilla, (Aralia Nudicaulis,) as neither its odor nor taste are those of the smilax. The stillingia and yellow dock are indigenous medicines, gaining favor with the profession; in this form they will be found very convenient. Lastly, the taraxacum was examined and found to be a saccharine liquid having the odor and taste of the root, but not manifest to the same degree as in the spirituous fluid extract, or the prepared juice.

Having thus hurriedly passed the several preparations of the Messrs. Tilden in review, it seems right in the connection to make a few remarks on this new branch of the enterprise of these gentlemen. With their gardens and apparatus described before (see vol. xxiii. p. 386,) they have great advantages for the preparation of the fluid extracts of indigenous plants; and for the same reason, with choice drugs, they may equally well prepare fluid extracts from them. There are some cases, however, where the apothecary should always prepare them himself, because so much depends on their uniformity, that he is not justified in relying upon a commercial article of which he cannot be assured of the age and condition. On the other hand, there are many which those gentlemen may produce with great advantage, especially to country practitioners, who often need concentrated medicines in their rural pharmacy. Necessarily more prone to decomposition than solid extracts, it is an important point to render them as permanent as possible, and to this end the propriety of an alcoholic menstrum is sometimes undoubted, even where its solvent power is not called into play.—*American Jour. of Pharmacy*, September, 1855, and *N. J. Med. and Surg. Reporter.*

*Hospitals—Historical Sketch.*—The following sketch of the early history of hospitals we extract from an interesting address by Joseph M. Smith, M. D., delivered on the occasion of the inauguration of the New South Building of the New York Hospital.—*Ed. Med. and Surg. Reporter.*

"The institution and multiplication of hospitals are among the most striking evidences of the progress of civilization, and of the benevolent and noble spirit of those engaged in their support and management. Their origin is distinctly traceable to the enlightenment and humanizing influences of Christianity. Among the ancient Greeks and Romans, hospitals were unknown. The poor were allowed to suffer and die under such shelters as might fall in their way. The wounded in battle had no retreat where they could collectively receive medical care. The temples were the only places where the sick congregated, not for the purpose of admission as resident patients, but for the purpose of obtaining a knowledge of the remedies which were there recorded as having been curative in cases similar to their own.

"In their general character, as sources of medical relief, the temples of heathen antiquity were more nearly allied to modern dispensaries, than to hospitals. It is said by a distinguished medical scholar, Professor Bartlett, in his *Discourse on the Times, Character and Writings of Hippocrates*, that 'The sick who visited the temples for relief, were subjected, under religious forms, to a preparatory regimen, consisting of prolonged abstinence, or a rigorous diet, and various purifying ablutions and inunctions of the body. After these preliminaries, a night was passed in the temples, and the patients subjected to the treatment ordered by the Asclepiades. A certain number of the patients who had been cured, left in the temples votive tables, grateful offerings to the gods, containing brief records of their diseases, and their cure.' Strabo says, 'the Temple of Epidaurus is always full of the sick, and of the tablets on which the treatment is inscribed; and it is the same at Cos, and at Tricca.'

"The epoch of the institution of hospitals is usually refered to the sixth century, or the reign of Justinian. They seem to have been for a long time connected with monasteries, and under the direction of those in whom were united the medical and clerical characters. But the greater number of infirmaries have arisen since the Reformation; and in many instances, the edifices now used as hospitals were formerly conventual establishments. Hundreds of years elapsed before institutions were founded for the special and exclusive benefit of the sick and poor. Indeed, as matter of history, hospitals scarcely deserve to be mentioned before the twelfth century, in which age St. Bartholomew's, in London, was founded by Henry I., though subsequently endowed by Henry VIII., and rebuilt in the reign of George II. Of the few of any note established in the fifteenth century, was Trinity Hospital, a small institution at Edinburgh. After this period the number rapidly increased. Among those which sprang up as general or special infirmaries, and which are the most celebrated and best known to us, in the sixteenth century, were St. Thomas', of London, and the enlargement of the Hotel Dieu at Paris, one of the oldest in Europe, dating back as far as the seventh century; in the seventeenth century, were the Bethlehem, St. Louis, La Pitie, La Charite, La Salpetriere; in the eighteenth century, St. George's, the London, Guy's, Middlesex, Lock, St. Luke's, Royal Infirmary of Edinburgh, Des Enfans Malades, and Des Veneriens. There were also many others founded in metropolitan, provincial, and colonial situations; and among these last, was one in the colony and city of New York. In the present century, infirmaries have so multiplied, that there are few places, especially in maritime countries, having a city population, in which there is not one or more established with chartered rights, or under the management of a municipal government."

---

### DEATH FROM CHLOROFORM.

We take the following from the Boston *Medical and Surgical Journal*, and hope it may contribute, in some degree, to lessen the recklessness with which this most dangerous agent is used.

"In our last number we noticed a case in which death occurred from the inhalation of chloroform recently in Edinburgh. We regret to state that the same accident took place in this city on Saturday, Jan. 5th. We copy from the Boston Journal the following statement of the case, as prepared by Dr. Emery, who administered the chloroform.

"Between the hours of 1 and 2 o'clock, on the 5th inst., I commenced to administer chloroform to Mrs. P. A. Morgan, at her request, for the purpose of removing some teeth. I commenced with a small quantity—should think from two to three drachms, on a sponge. She inhaled it without difficulty for a minute or two. Her pulse was not strong, but uniform. She then commenced to be excited, and said that I was going to extract her teeth, and she should know all about it. She said that Mrs. Paige, (the lady who accompanied her) was getting the forceps to extract them with. I think about one minute had passed during this conversation and excitement. I then removed the sponge from her mouth, and in a few moments she became quiet, and satisfied that there had been no attempt made to remove her teeth. In a few moments I commenced the operation again with the same amount of chloroform. She inhaled it without difficulty about as long as she did before, and became so much excited that she got up out of the chair and insisted that I had extracted her teeth. She spit on the floor and looked to see if it was blood, and she insisted that some one was coming into the room whom she did not want to see. I sat her down in the chair again, and she then went into a spasm, closed her teeth, and breathed with difficulty. I sprinkled water on her face, and the muscles relaxed, and I asked her to get up and we would place her on the lounge.—She made an effort to rise, and with my assistance stood on her feet, and then instantly sank to the floor. With the assistance of Mrs. Paige, I placed her on the lounge, and then there was a rush of blood to the brain. I sprinkled water in her face again, but she showed no signs of being conscious. Mrs. Paige went for assistance, and I immediately commenced artificial respiration by insufflation, and kept it up until Dr. Stedman came in, which was but a few moments." To this account by Dr. E., the Journal adds—

"As was stated in our paper yesterday, the inquest was held by Dr. C. H. Stedman, and the jury returned the verdict 'that the deceased came to her death from the effects of the chloroform, and that the chloroform was a pure article, and was given at the urgent solicitation of the deceased, and with all proper care and discretion.' They further say, 'from the testimony and opinion of medical experts in this case, the jury feel compelled to caution the public against the use of chloroform, as being a dangerous anæsthetic agent.' "

With this recommendation we entirely agree, and we have before urged, not the necessity of caution, (for caution seems to be of no avail in these cases,) but the abandonment of chloroform and concentrated chloric ether, as anæsthetic agents, in ordinary cases*; the more especially since we have the original article used for producing insensibility to pain, sulphuric ether, which is efficient, cheap, and above all, safe. We are not aware that any case of death has occurred from the direct effect of the inhalation of ether, and although it is possible that such an event may take place, the article is beyond all question more safe than chloroform, the number of deaths from which now amounts, we fear, to thousands.

We cannot help thinking that the amount of chloroform used in this case was very large. It appears that from "two to three drachms" were first inhaled, and that the same amount was repeated. We believe that the most approved practice in England is to pour a few drops (twenty minims, Druitt) on a

---

*To which we add our earnest support.—Editors.

handkerchief folded into a hollow cone, or into an apparatus specially designed for the purpose, and held at the distance of a few inches from the patient's nose. This is to be repeated occasionally until anæsthesia is produced; in many cases a single drachm is sufficient.

---

*Medical Journals—Should the Young Practioner Read them?*—A well-regulated medical periodical is the medium of communication of the experience, thoughts, and reflections of medical men to one another, and of the important discoveries in the different departments of medicine. They supply on a large scale, in a certain degree, the place of societies and associations for improvement, by affording the members of a profession, widely-scattered, a medium for the rapid interchange of views, and for the discussion of principles and methods of practice. While activity in the cultivation of the various branches of medicine is necessary to their healthy existence, they reciprocally tend to stimulate the growth and development of these branches, by the wide-diffusion of recent discoveries among earnest laborers in these fields of research.

A serial publication thus becomes, not only a record of the progress of medical science, but also a great repository of facts, valuable for future study. It is the inexhaustible store-house, from which the compilers of practical treatises obtain the materials for their works.

The high-toned liberal and independent medical periodical is essential, also, to the ethics of the profession. It creates and maintains a sentiment adverse to the petty jealousies and envious strifes, which too often arise between individual members, and totally subversive of all the low arts by which the charlatan imposes upon public credulity.

But the laborious country practitioner can best appreciate the well-regulated periodical. Isolated from all professional associations, he is deprived of the satisfaction of communicating his doubts and perplexities to others, and from mutual discussion deriving light and assistance. His position affords him but limited advantages and time for study and careful investigation; while, from the instructive field of pathology he is entirely debarred. To this large and most respectable class, the medical press is a desideratum. It brings them in close communication with their brethren, scattered over the civilized world, and makes them participants of whatever learning, research, or experience can afford. It spreads before them, for contemplation, the precepts of the public teachers of their profession, the discoveries of the microscopist and pathologist, and the experience of the hospital physician and surgeon.

We are led to these remarks by the annually repeated advice of a prominent medical teacher, to his graduating class, never to read medical periodicals. "If you have leisure," he is accustomed to say, with characteristic emphasis, "and must read, select novels instead of medical journals, for your instruction"

We can best prove the injustice of this slur upon medical journals, and show its ill effects upon the young practioner who has the simplicity to follow it, by making an extract from a letter just received from a young physician, located in the interior of an adjoining State. He says, "I settled in this place about two years since, under the shadow of an old physician, who had long monopolized the practice of the town. During the first year and a half I had nothing to do; but, undaunted, applied myself diligently to a careful review of my studies, and the perusal of the best medical periodicals. The opportunity finally offered for me to apply my knowledge to good account. I was called to visit a young man with a dislocated femur, which the old physician, my rival, had in vain attempted to reduce with pullies, after torturing the patient several hours, to the horror of the bystanders. Before visiting the patient, I carefully reviewed the admirable, and to me invaluable paper of Dr. Markoe, in the the N. Y. Journal of Medicine, on reducing the dislocated femur by manipulation. I found the patient and friends alarmed, and fearful of having instruments again used. I placed him in the proper position for manipulation, and, in the presence of a multitude of bystanders, began the required movements of the limb. Without causing the slightest pain, I carried it through the proper circle, and was about to bring it down, when the head slipped gently into its socket, to the great relief of the patient, and the satisfaction of the friends. I need hardly add, that within one month of that operation, I had all the business I could do."

We could exhibit many other facts of a similar nature, from our correspondence, illustrating the importance of medical journals to the practitioner, but it would be a supererogatory task. Their value is indisputable to every lover of his profession, and it is to this class we address ourselves.—*N. Y. Jour. of Medicine.*

---

*External Employment of Oil of Turpentine in Consolidated Lungs from Pneumonia.*

One of the most interesting features we have recently noticed in Dr. Todd's practice, at King's College Hospital, is a plan of treating solidified lungs and strumous pneumonia by turpentine—a mode not new, possibly, but eminently valuable, and one in which Dr. Todd seems to gain greater confidence every session. If we dwell on minor points of this character, it is because we see such cases too often overlooked, as they present themselves in the out-patients' department of hospitals, and because this medicine has been the secret of many reputed cures of consumption amongst practitioners, outside the profession.

J. B——, aged twenty-one, was admitted into King's College Hospital, October 2nd, suffering under various symptoms of chest disease, the result of a severe attack of pneumonia. Dr. Todd pointed out to his class that the entire lung of one side was completely solidified. This lung had, in all probability, gone through the three stages of pneumonia so familiar in practice, but often so very unmanageable in their results, the first and second stages of pneumonia inflammation usually merging into one another, and leaving the lung quite solid.

The first, or congestive stage, is easily cured, when detected early, as we recently took occasion to show in cases in the practice of Dr. Willshire and Dr. Parkes. The second stage, in which these congested vessels relieve themselves by fibrinous exudation into the lung, and slight hemorrhage, as shown in the characteristic rust-colored expectoration, is also very curable. It is not very well named, perhaps, as hepatization, in which the lung is sprinkled sometimes with pinky granulations, and which Bayle and others look upon as the first stage of tubercular disease. Dr. Todd also described the exudation into the parts, with its granular blastema, blood-corpulses, and exudation-cells, the latter, perhaps, traceable from the state of granular corpuscles to that consolidated stage where we now meet it, or the third stage of this disease—that of grey hepatization, with its various mottlings or spotting of the lungs. A purulent fluid sometimes exudes, yet this is not essentially a state of suppuration, though very nearly allied to it; it is also different from tubercle, though in many cases next door to it—the chief practical point, to Dr. Todd, being, that the consolidation of pneumonia does not necessarily destroy the vesicular structure of the lung, no more than effusion into the iris, in iritis, removable by mercury, destroys the structure of that delicate part; the inflammatory effusion, in pneumonia, occurring almost universally through the lung tissue, as well as *into* the air cells, and the intervesicular tissue. In a very advanced stage of pneumonia, it is true, we may have pulmonary abscess. This was evidently not the case in the present instance. The treatment adopted by Dr. Todd, and which he finds most effectual, was the following: Wine, six ounces, daily; a draught every third hour, composed of julep of acetate of ammonia, with aromatic spirits of ammonia in excess; and strong turpentine stupes, carefully applied, every night and morning, over the back of the chest and site of the consolidated lung. Diet moderately stimulating.

October 4th.—On seeing this patient two days after, we found he had already begun to improve; the lung, previously quite dead, as far as respiratory murmur was concerned, began to give signs of healthy vesicular murmur. The right lung, as will have been observed, is most usually that affected. Dr. Tood has great faith in the stimulant action of turpentine —a remedy not often used, but which in this and numerous other cases has proved almost specific in its action. In phthisical cases, also, it may be used, combined with strong acetic acid, when its action becomes even still more beneficial.

25th.—This man is quite well.—*Lancet*.

---

*New Remedies.*—At the London Hospital, a case of syphilitic warts has improved under a lotion of decoction of tormentiall.

A case of fracture, ununited for four months, probably from effects of scorbutic disease, improving under the use of lemon-juice.

Equal parts of collodion and perchloride of iron—collodion, Venice turpentine, and castor oil, as impenetrable coverings for the cure of local inflammation, are spoken about.

A watery extract of belladonna is used in Italy instead of secale cornutum, for producing relaxation of the os uteri; it is said to act in the same way, not by paralyzing the muscular fibres, but by stimulating them, a function denied to secale in that country.—*Lancet*, Feb., 1855, from *Boston Med. & Surg. Journal*.

*Lard as an Antidote to Strychnia.* By G. W. Thomas, M. D., of Camden, N. J.

Having seen an article in the *Journal of Pharmacy,* written by a physician of Virginia, detailing some experiments, which he thinks proves lard an antidote to strychnia, I was induced to repeat his experiments. I at first (acting upon the suggestion of the Editor of the *Journal,*( tried olive oil. The cats all died in a period of time varying from five to fifteen minutes. I then tried the lard; it also failed. One of the cases, a large poodle dog, after taking a half grain of strychnia, *ate two pounds of lard.* Convulsions came on in thirty minutes, fearfully violent; subsided and came on again in an hour, and a few hours after he was found *dead.* This case may seem to have had its end postponed by the antidote(?). But another dog, of equal size, which took the same quantity of the poison and *no lard,* lived twenty-four hours, with occasional convulsions, before he died.

The correspondent of the *Journal* was sure his strychnia was a good article. I am equally certain that *my lard* was simon pure, as I procured it from Mr. George House, druggist, in this city.

I would ask the question—are not medical correspondents generally *rather* hasty in bringing new remedies and antidotes before the public?

The experiments were made immediately upon the appearance of the article in the *Journal.* And I deferred making them public, hoping some other physician would give his experience with the alledged antidote.—*N. J. Med. and Surg. Reporter.*

---

*Card of the Committee on Prize Essays of the American Medical Association.*—At a meeting of the American Medical Association, held in Philadelphia, May, 1855, the undersigned were appointed a committee to receive voluntary communications on medical subjects, and to award prizes in accordance with the regulations of that body.

Each communication intended to compete for a prize must be addressed to the Chairman of the Committee, at Ann Arbor, Michigan, before March 20th, 1856, and must be accompanied by a sealed packet, containing the name of the author, and marked, exteriorly, by a sentence or motto corresponding with one upon the essay, which packet will not be opened unless the essay belonging to it is successful in obtaining a prize.

Unsuccessful papers will be returned, on application after the adjournment of the meeting of the Association, in Detroit, in May next.

A. B. Palmer, M D., (Chairman), Samuel Denton, M. D., A. R. Terry, M. D., A. Sager, M. D., S. H. Douglass, M. D., C. L. Ford, M. D., E. Andrews, M. D.—*N. Y. Jour. of Med.*

*Solution of Tannin in Aromatic Sulphuric Acid as a Hœmostatic.*—In a late number of the *Medical Counsellor*, Dr. A. B. Butler, of Clifton, Ohio, recommends the above combination in hemorrhage from the various organs. Several cases of menorrhagia have been relieved by it without a single failure. The following is the formula recommended:—

℞ Acid. tannici..................................ʒiv.

Acid. sulph. arom..............................f℥j. M.

Dose, fifteen drops three times a day, or more frequently, if the urgency of the symptoms require it.

Some chemical changes that take place are worthy of notice. When the mixture has stood for some time, there is a slight deposit of a buff-colored sediment, which is, probably, sulphate of tannin formed by the direct union of small portions of the two acids. The remainder of the tannic acid, by the acquisition of more oxygen, is converted into gallic acid, so that the combination is really a mixture of gallic and sulphuric acids, diluted, in which is deposited the small portion of sulphate of tannin already referred to.

In the bowel complaint of children—chronic cholera infantum—Dr. Butler has, after premising a few powders consisting of minute portions of calomel, ipecacuanha, and opium, for the fever, found great satisfaction in the use of the following combination for the purpose of restoring the bowels to a healthy condition :—

℞. Acid. tannici................................ʒj.

Acid. sulph. arom.....................fʒij.

Tinct. opii camph.....................f℥ss. M.

Dose, ten to twenty drops to a child, during the first dentition, every three hours, until the disorder is checked, afterwards less frequently, to keep up the impression until the natural healthy action is restored.

The opium in this preparation, notwithstanding its incompatibility with gallic acid, retains enough of its anodyne properties to make it a valuable adjuvant.—*N. J. Med. and Surg. Reporter.*

---

*Caustic Pulverizer;* by R. H. Thomas, M. D.—This is an apparatus consisting of a mahogany box, two wheels and a "grindstone." The purpose of it is to reduce the caustic to an impalpable powder for inhalation by the patient. The inventor speaks favorably of this method of applying the nitrate of silver to the air passages.—*N. Y. Jour. of Med.*

*Acid Beef-Tea.*—The following is the formula for an acid beef-tea, which Mr. Paget has recently introduced into use in St. Bartholomew's Hospital. It was originally suggested by Leibig, and is intended, in cases of great debility, to supply the stomach with fluid nutriment, which, containing its own acid, will task the digestive powers in the least possible degree.

Take of beef, veal, or chicken, chopped fine, half-a-pound,
" of hydrochloric acid (strong) four drops,
" of water (cold) eighteen ounces,
" of common salt, a pinch.

After macerating for an hour, strain off the fluid, using no pressure. The remaining meat may be treated with half-a-pint of water, and a second solution obtained. If the fluid be not clear a second straining will be needed. The solution does not taste acid, and is very palatable. Pepper or other spice may be added, according to the patient's taste.—*N. Y. Journal of Med.*

---

### TO CORRESPONDENTS.

As the Editors find it very inconvenient to attend to the Financial department of the Journal, correspondents upon business not immediately connected with the Editorial department, will confer a favor by directing their communications to J. G. Westmoreland, M. D., Dean of the Faculty of the Atlanta Medical College.

---

### SUBSCRIPTIONS RECEIVED.

D. Kendall, Ga.; W. Hartsfield, Ala.; S. H. Freeman, Ga.; J. Bivins, Ga.; Robt. A. Johnson, Ga.; Geo. N Jones, Ga.; Wm. A. Taylor, Miss.; M. B. Bogan, Ga.; Thos. Denny, Ga.; John Rhea, Ga.; W. P. Parker, Ga.; T. M. Darnall, Ga.; P. M. Tidwell, Ga.; T. P. Burgamy, Ala.; J. M. Babee, Ga.; J. Coston, Ga.; M. G. Slaughter, Ga.; Jas. Thweat, Ga.; S. H Dean, Ga.; J. H. Ragan, Ga; M. A. Leak, Ga.; J. Hillsman, Ga.

